# A serological survey of pathogens associated with the respiratory and digestive system in the Polish European bison (*Bison bonasus*) population in 2017–2022

**DOI:** 10.1186/s12917-023-03627-y

**Published:** 2023-06-01

**Authors:** Anna Didkowska, Daniel Klich, Magdalena Nowak, Marlena Wojciechowska, Kinga Prolejko, Ewelina Kwiecień, Magdalena Rzewuska, Wanda Olech, Krzysztof Anusz

**Affiliations:** 1 Department of Food Hygiene and Public Health Protection, Institute of Veterinary Medicine, Warsaw University of Life Sciences (SGGW), Nowoursynowska 159, Warsaw, Poland; 2 Department of Animal Genetics and Conservation, Institute of Animal Sciences, University of Life Sciences (SGGW), Ciszewskiego 8, Warsaw, 02-786 Poland; 3 Department of Preclinical Sciences, Faculty of Veterinary Medicine, Warsaw University of Life Sciences, Ciszewskiego 8, Warsaw, 02-786 Poland

**Keywords:** Bluetongue Virus (BTV), Bovine coronavirus (BCoV), Bovine herpes virus type 1 (BoHV-1), Bovine viral Diarrhea Virus (BVDV), ELISA, European bison, Infectious bovine rhinotracheitis (IBR), Pestivirus, *Mycoplasma bovis*, Serology, Wildlife

## Abstract

**Background:**

The European bison (*Bison bonasus*) is a near threatened species and requires health monitoring. The aim of the present study was to determine the prevalence of antibodies to pathogens known to cause respiratory and digestive illness in ruminants.

**Results:**

In the studied 328 European bison, the highest seroprevalence was observed for Bovine herpesvirus-1 (BoHV-1) (50.27%), Bovine Coronavirus (BCoV) (26.36%), and Bluetongue Virus (BTV) (12.83%). For *Mycoplasma bovis* strains and Bovine Viral Diarrhea Virus (BVDV), positive results were rare. Interestingly, a higher prevalence of BTV antibodies was noted in the northeastern populations and older animals.

**Conclusions:**

Our findings indicate that the Polish European bison population appears to have considerable contact with BoHV-1; however, this does not appear to be of great significance, as clinical symptoms and *post-mortem* lesions are rarely noted in Polish European bison population. The high seroprevalence of BTV in the north-east of Poland is an ongoing trend, also noted in previous studies. It is possible that European bison may perpetuate the virus in this region. This is the first report of antibodies for BCoV in European bison.

**Supplementary Information:**

The online version contains supplementary material available at 10.1186/s12917-023-03627-y.

## Background

The European bison (*Bison bonasus*) is listed as a near threatened species in the International Union for Conservation of Nature’s (IUCN) Red List of Threatened Species [1], and therefore requires active conservation measures, including health monitoring [2]. One of the easiest, and most suitable, ways to monitor exposure to pathogens in wildlife is serological monitoring. Although a number of infectious agents that can potentially cause reproductive disorders in European bison have been identified [3], limited data currently exist about those that can affect the respiratory or digestive tract [4]. While studies on diseases of the respiratory system in European bison in Poland have focused on tuberculosis [5], other pathogens also present a threat. For example, diarrheas of viral origin might result in high mortality in cattle [6–8] and should be also monitored in European bison populations. Therefore, there is a need to determine the degree of current, or recent, exposure to a range of respiratory and gastrointestinal pathogens in European bison herds that are also prevalent in domestic cattle.

One particularly significant bacterial species causing respiratory diseases in cattle is *Mycoplasma bovis.* Not only is it a major cause of bovine respiratory disease (BRD) complex, but also has an important role in other diseases such as mastitis, keratoconjunctivitis and arthritis [9]. However, while infections are often asymptomatic in cattle [10], several high-mortality epizootics have been noted in American bison (*Bison bison*) [11]. Considering the close genetic relationship between European and American bison, this is clearly a significant pathogen that should be monitored.

Another important pathogen is bluetongue virus (BTV), which causes a re-emergent insect-transmitted disease: bluetongue. Although the disease was previously confirmed in regions with warmer climates, BTV appears to have spread into Europe over the past 15 years, resulting in economical losses and the implementation of BTV vaccination programs in some regions [12]. The clinical signs and mortality rates are variable and depend on *inter alia* serotype, the amount of virus, host species and certain environmental influences [13, 14]. One of the clinical signs of BT is respiratory distress [15]; however, the clinical form is mainly noted in sheep [14]; indeed, wild European moufflons (*Ovis aries musimon*) have been found to demonstrate inflammation of the mucous membranes, swelling and hemorrhages [16]. Free-living ruminants might serve as vectors and play a role in virus maintenance in Europe, with red deer (*Cervus elaphus*) being considered a reservoir [13]. It is, however, important to monitor the spread of BTV in European bison; this is as borne out by the fact that BTV-8 caused the deaths of 10 of 33 bison in the Hardehausen (Germany) Breeding Center in 2007 [17]. Moreover, with the continuing advance of global warming, and considering the sensitivity of wild ruminants to infection [18], there is a need to control this pathogen in the Polish European bison population, particularly since the antibodies have previously been noted in this species [19].

Bovine herpesvirus-1 (BoHV-1) is associated not only with respiratory clinical symptoms (IBR – infectious bovine rhinotracheitis) but also with pustular vulvovaginitis (IPV) and infectious pustular balanoposthitis (IPB) [20]. The disease is usually not life-threating and animals are often lifelong reservoirs of the latent virus. It is known to affect some endangered bovine species like mithun (*Bos frontalis*) and yak (*Poephagus grunniens*) [21]. The virus is endemic worldwide, including Poland: studies found the herd-level seroprevalence to be 53% in non-vaccinated dairy cattle herds with clinical respiratory symptoms [22].

Two viruses associated mainly with diarrhea symptoms in cattle are bovine coronavirus (BCoV) and bovine viral diarrhea virus (BVDV). BCoV is known to cause neonatal calf diarrhea, winter dysentery and shipping fever in adult cattle [6]. BCoVs and bovine-like coronaviruses have also been confirmed in various wild ruminants, including water buffalo (*Bubalus arnee*), llamas (*Lama glama*), alpacas (*Vicugna pacos*) and wild goats (*Capra aegagrus*) [6]. BVDV, of the genus Pestivirus, is responsible for significant economic losses in cattle farming worldwide, being a cause of both diarrhea and infertility in cattle [23]. BVDV has been confirmed in numerous wildlife species [24], and some, such as white-tailed deer (*Odocoileus virginianus*), are considered to be reservoirs of BVDV [25]. The potential for pestiviruses to spread between livestock and wildlife is still being discussed [24, 26].

The aim of this study was to evaluate the current threat posed by one bacterial (*M. bovis*) and four viral pathogens (BTV, BoHV-1, BVDV, BCoV) on the respiratory and digestive system in European bison based on a serological study, and to indicate future directions for monitoring the health of European bison.

## Results

### ELISA results

The ELISA results indicated the following percentages of positive animals: *M. bovis* 3.33% (6/180), BTV 12.83%, coronavirus 26.36% (34/129). For IBR antibodies, 50.27% (90/179) animals were positive = and 14.52% (26/179) were suspect. For BVDV, these values were 6.99% positive and 3.23% suspect (6/186).

### Statistical analysis

#### BTV

**Fig. 1:**
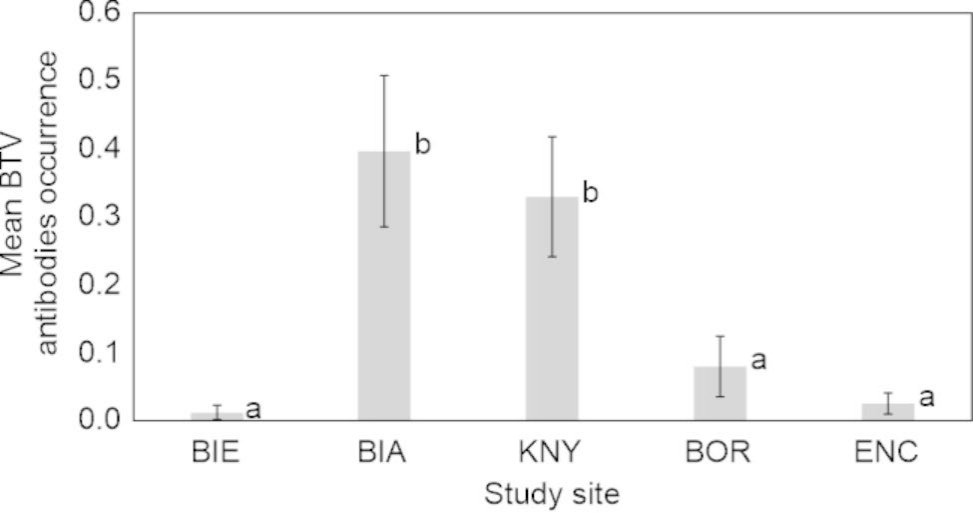
Mean (± SD) Bluetongue virus (BTV) antibodies occurrence, evaluated using commercial ELISA, in European bison in generalized linear model and pairwise comparison in LSD test (BIE: Bieszczady Mountains, BIA: Białowieska Forest, KNY: Knyszyńska Forest, BOR: Borecka Forest, ENC: animals in enclosures); similar letters (**a**, **b**) indicate pairs not different in statistical pairwise comparison. The Białowieska and Knyszyńska forests presented a high seroprevalence, which differed from that of other wild populations and animals in enclosures

Both age and study site significantly explained the presence of antibodies against BTV in European bison (χ^2^ = 89.45 df = 6, p < 0.001, Supplementary Table 1). BTV frequency in European bison varied considerably across the study sites. The Białowieska and Knyszyńska forests presented a high seroprevalence, which differed from that of other wild populations and animals in enclosures (Fig. 1).

**Fig. 2:**
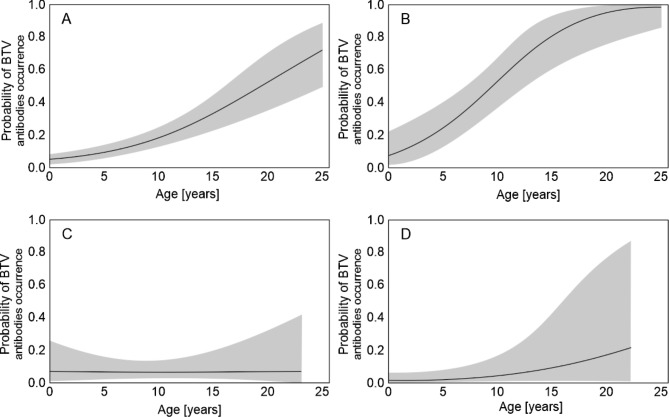
The risk of Bluetongue virus (BTV) antibodies occurrence, evaluated using commercial ELISA, in European bison in: **A**) all analyzed animals, **B**) animals in Białowieska and Knyszyńska forests, **C**) animals in Bieszczady and Borki forest and **D**) animals in enclosures. Within populations with high prevalence (Białowieska and Knyszyńska forests), a similar increased risk of seropositivity i.e. over 50%, was observed at an age of 10 years. In the low-risk free-ranging populations (Bieszczady and Borecka forest) and in enclosures, no significant elevation in the risk of BTV antibodies occurrence was observed with age

The European bison were more likely to be seropositive with BTV with age. Among the entire sample, the older individuals, i.e. those over 17 years of age (Fig. 2A) were found to have more than a 50% risk of contracting BTV. However, in populations with high seroprevalence (Białowieska and Knyszyńska forests), a similar increased risk of seropositivity, i.e. over 50%, was observed at an age of 10 years (Fig. 2B). In contrast, in the low-risk free-ranging populations (Bieszczady and Borecka forest) or those in enclosures, no significant elevation in the risk of BTV antibodies occurrence was observed with age (Fig. 2C, D).

#### IBR and Coronavirus

The occurrence of antibodies to the BoHV-1 and BCoV in European bison was not dependent on age or sex, and did not differ between individual study sites (Supplementary Tables 2 and 3). Both models were statistically insignificant (for BoHV-1 – χ^2^ = 1.96, df = 6, p = 0.923; for BCoV – χ^2^ = 6.21, df = 6, p = 0.400).

## Discussion

The present study describes a large-scale cross-sectional survey of the seroprevalence of selected pathogens in European bison in Poland. Its findings highlight general trends in selected pathogens and indicate those which can pose a current threat for European bison. It should be emphasized that performing a study on such a large number of individuals of a protected species required a lot of effort.

Our finding indicates that the occurrence of antibodies to *Mycoplasma bovis* and BVDV is rather low, while the seroprevalence of BoHV, BoCoV and BTV is higher. It is possible that in some regions, European bison may be responsible for the maintenance of BTV in the sylvatic cycle [19, 27]; however, it must be emphasized that further research is required to confirm this. Additionally, some of tested pathogens (BTV, BoHV-1, BVDV) may have immunosuppressive effects and thus increase the exposure to other infections, such as tuberculosis. An important consideration is that the possibility of direct or indirect contact with livestock, e.g. by sharing common pastures, which is particularly likely in the Białowieska Forest and Bieszczady Mountains [28], may contribute to the circulation of pathogens in the environment. Hence, the fact that co-infections were not taken into consideration can be regarded as a limitation of this study.

It should be stressed that no increase in the number of pathological lesions, such as those indicating previous pneumonia or previous enteritis, was observed in the respiratory and digestive systems, in European bison during monitoring necropsies (based on annual reports from the project: Comprehensive monitoring of European bison population and habitat, unpublished). In addition, the occurrence of clinical symptoms from the respiratory or digestive system remains low; as such, high BoHV-1 seroprevalence should continue to be monitored, but it is unlikely to be an emerging problem in European bison at present.

Our data indicate a low seroprevalence of *M. bovis*. It is important to emphasize that while the seroprevalence is quite high in Polish cattle (76.7%) [29], *M. bovis* infections are typically asymptomatic [10]. This difference might be based on the virulence of the strain, which would be influenced by the host species [30]. Interestingly, a study from western Canada found that up to 79% of American bison herds have more than one seropositive member [31]. However, it is important to note that several outbreaks have been reported in *Bison bison* in North America, with mortality rates as high as 45% [11], and as such, there is a strong need to monitor the disease in European bison.

Even though *M. bovis* infection is rare in free-living animals, it should nevertheless be included as a differential diagnosis for pneumonia in wildlife [32]. Based on our findings and those of previous studies, we recommend that European bison with pneumonia should also be tested for *M. bovis*, together with tuberculosis and other respiratory pathogens, especially when outbreaks of pneumonia occur. So far, antibodies against *Mycoplasma bovigenitalium* have been confirmed in European bison with balanoposthitis [33]. Previous serological studies have revealed a similar low seroprevalence of *M. bovis* in European bison [4, 34].

As the BTV occurring in Poland has been determined as serotype BTV-14 [35, 36], we suspect that the antibodies detected in this study were also against BTV-14. This type has also been confirmed in Lithuania, Latvia, Estonia, and Russia [37–39], which could explain its presence in northeastern Poland. It has also been confirmed in this area of Poland in both livestock and wildlife [19, 35, 36]. However, as positive animals are asymptomatic, the strain present in the environment can be thought of as attenuated and vaccine-like, as it has occurred almost in the same time in Russia which is not typical. BTV-14 is similar to a South African vaccine strain which demonstrated only mild clinical signs in experimentally-infected sheep [40]. Hence, it has been suggested that BTV vaccine may have been used illegally in the past. [41].

The occurrence of BTV-14 in northeastern Europe is probably not connected with the BTV-8 epidemic in western Europe [42]. BTV-8 infections in European bison have been described, with mortality rates as high as 20% [43]. The higher seroprevalence of BTV in older animals is in agreement with previous reports [19, 44–46] and can be explained by longer exposure time. Nevertheless, our finding that only animals of over 17 years old are at serious risk of BTV seropositivity can be misleading, as the age-associated increase in antibody titer depends on the occupied area. In populations with a high seroprevalence animals aged 10 years and older demonstrate a greater than 50% chance of being BTV-seropositive. Higher seroprevalence has been detected in the Knyszyńska and Białowieska Forests which can be associated with environmental conditions: forests with wet grounds are favorable habitats for *Culicoides* to breed and produce multiple generations [46].

Even though BoHV-1 is mostly spread in cattle, it has been also detected in wildlife [47–50]. While some European countries have managed to eradicate IBR, Poland is not one of them [51]; indeed, antibodies have been found in livestock and wildlife in Poland [52–56]. In the present study, about half of the tested European bison were seropositive for alphaherpesvirus, indicating that BoHV-1 infections are present among both free-ranging and captive European bison in Poland. The serological survey, involving the largest number of individuals, for IBR in European bison, indicated 13.3% (8/60) BoHV-1 seroprevalence; however, the survey was limited to the Białowieska Forest population [53].

In Poland, the seroprevalence of BoHV in dairy cattle was found to range from 37.7 to 73.7% [22, 57], and a recent study on cervids found higher seroprevalence in captive animals than free-ranging ones [52]. No such tendency was demonstrated in the tested European bison in the present study. Considering that the European bison is highly sensitive to tuberculosis [5] it is worth mentioning that novel alphaherpesvirus has recently been isolated from a South American sea lion (*Otaria byronia*) with pulmonary tuberculosis [58]. In general, the health condition of the European bison population in Poland is good, so it does not seem that IBR is a problem for them at present. Subclinical BoHV infections are probably also common in this species as in cattle [59]. However, the situation should be monitored by continuing serological surveys and observing the general health of the population, with special attention to typical clinical syndromes for BoHV-1 infections including respiratory disease, genital disease, and late-term abortions [60].

Our findings serve as the first indication of the presence of BoCoV antibodies in European bison. It should be highlighted that contact with this virus is not incidental, with the seroprevalence in the tested animals being 26.36%. Bovine-like CoVs, i.e. host-range variants of BCoV, are crossing the interspecies barriers, and transmission between cattle and wild ruminants allows the virus to persist in the wild [61]. In this sense, European bison and other wild ruminants could potentially play a role in future epidemics and the evolution of the virus due to their continuous movement to seek new pastures.

The tested European bison demonstrated higher BVDV seroprevalence (6.99%) compared to a previous report, in which 0.8% had antibodies [4]. This shows an upward trend of possible exposure to this virus. However, the seroprevalence in this study was lower than reported 20 years ago [55]. Antibodies to BVDV were also detected in European bison from Whipsnade Wild Animal Park (UK) [62]. Due to the immunosuppressive nature of the virus, acute infections are often accompanied by comorbidities, typically manifesting as respiratory disease. Persistent BVDV infections in cattle lead to death after developing mucosal disease [63]. Hence, it is crucial to monitor the presence of immunosuppressive pathogens in European bison, due to their greater sensitivity to tuberculosis [64–66].

## Conclusions

In the studied European bison, the highest seroprevalence values were detected in the case of BoHV-1 (50.27%) and BCoV (26.36%) and BTV (12.83%). For *M. bovis* and BVDV, positive results were rarer. Interestingly, a higher prevalence of BTV antibodies was noted in the northeastern populations and older animals, which is consistent with reports in previous studies. Even though it has been shown that the Polish European bison population is in broad contact with BoHV-1, it does not appear to have a great influence on their health, and no clinical symptoms are generally noted. This is the first report to confirm the presence of antibodies for BoCoV in the European bison population.

Although we did not test for presence of viruses and bacteria, our findings indicate probable exposure to important bovine pathogens which can inform future surveillance efforts in European bison.

## Methods

### Materials

The blood samples used in this study were acquired opportunistically from a banked sample collection; this was obtained from animals that had been immobilized for standard veterinary care, for placing radio collars, or were culled or found dead. All live animals handling was performed under standard veterinary medical care activities, and therefore did not require approval according to the II Local Ethical Committee For Animal Experiments in Warsaw. The monitoring was carried out by local institutions responsible for European bison management, and each cull was performed with the necessary permit. The collection and storage of serum samples from dead animals were based on the decision of the Regional Director of Environmental Protection in Warsaw. According to the decision, the collection of dead animals for scientific purposes does not need any permit as described before [67]. From immobilized European bison, the blood was collected into sterile 6 ml tubes with a clot activator from the jugular or tail vein. From dead individuals, it was collected from the jugular vein, heart or body cavities. The samples were transported to the laboratory at 4^o^C. The tubes were then centrifuged, the serum was separated and stored at -20^o^C until serological tests were performed.

**Fig. 3:**
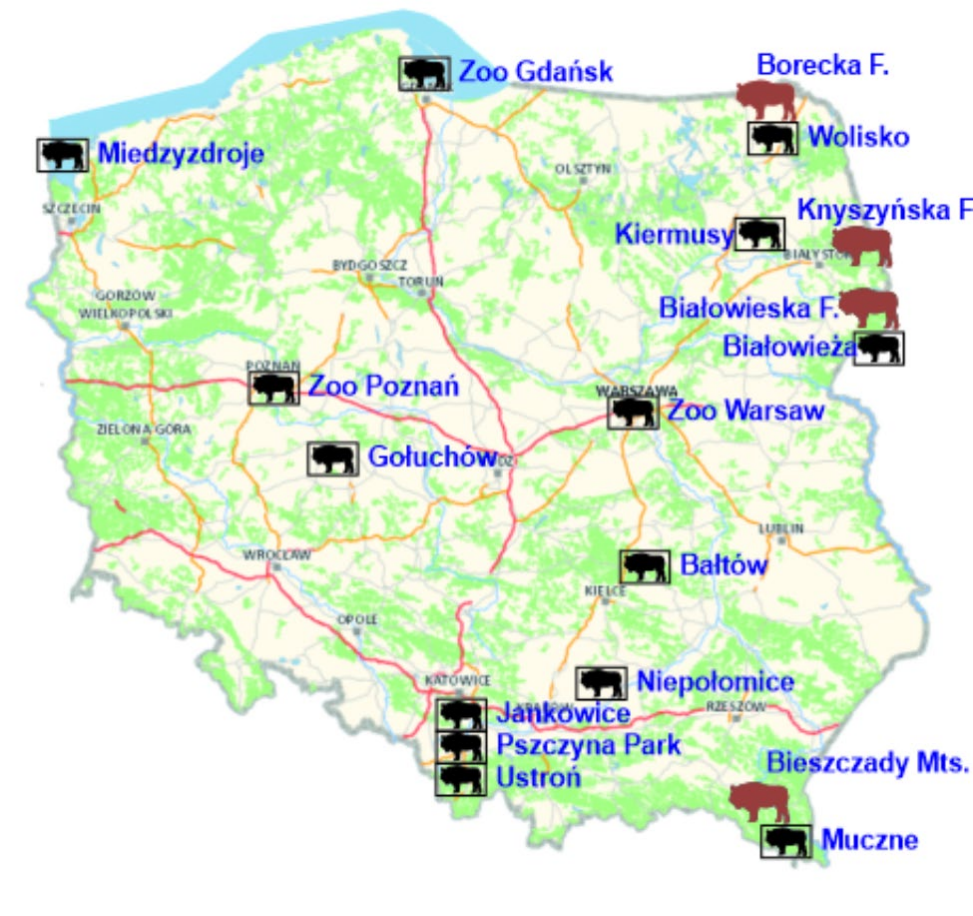
Location of study sites. The source map was obtained from the Geoportal.gov

Blood samples represented 328 European bison (181 females and 147 males) between October 2017 and February 2022. The samples came from four free-ranging populations located in the Białowieska Forest (n = 37), Borecka Forest (n = 41), Knyszyńska Forest (n = 41), and Bieszczady Moutains (n = 57). Among the captive herds, most of the samples were collected from Białowieża (n = 35), Muczne (n = 17), Niepołomice (n = 19), Pszczyna Park and Jankowice (n = 45), and various other captive herds (Bałtów, Wolisko, Gołuchów, Kiermusy, Międzyzdroje, Ustroń, Gdańsk Zoo, Warsaw Zoo, Poznań Zoo) (n = 36) (Fig. 3.). The age of the tested animals ranged from three months to 25 years.

### ELISA

Serum samples were allowed to thaw at room temperature and then tested with indirect enzyme-linked immunosorbent assay (ELISA) kits against specific antibodies to *M. bovis*, BTV, BoHV-1, BVDV and BCoV. All commercial ELISA kits were used in accordance with the manufacturer’s instructions. The results of each ELISA were read using an EPOCH spectrophotometer (BioTek Instruments Inc., US) at a wavelength of 450 nm and calculated following the instructions as for cattle. The positive and negative samples were taken from commercial kits. The positive/negative cut-off was used as for cattle, as per the manufacturer’s recommendations for each ELISA.

*M. bovis* antibodies, were detected using Monoscreen Ab ELISA *Mycoplasma bovis* kits (Bio-X Diagnostics S.A., Belgium) (n = 180). Briefly, after dilution, the samples were added to the wells sensitised by a recombinant protein from *M. bovis* expressed by *Escherichia coli*. Subsequently the plate was incubated and washed, and conjugate (protein G peroxidase-labelled) was distributed to the wells. Following a second incubation and washing, the chromogen (tetramethylbenzidine) was added. If specific antibodies were present in the sera, the conjugate remained bound, and the enzyme catalysed a colour change from colourless into a pigmented compound. Finally, a stop solution was added to each well. The intensity of the resulting yellow colour was proportionate to the titre of the specific antibodies in the sample.

INgezim BTV DR (Inmunologia y genetica aplicada, S.A., Spain) (n = 304) kits were used to detect antibodies specific for BTV. The kit is intended for sheep, goats, and cattle and can detect a low level of antibodies in the sera of infected and vaccinated animals. The plates are coated with VP7 protein of BTV, which binds any BTV antibodies in the serum. After sample distribution and incubation, the plates were washed, and colourless conjugate added to detect conjugated antibodies. Following another incubation period and washing, the chromogenic substrate (TMB − 3,3’,5,5’-Tetramethylbenzidine) was added. After stopping the reaction, the intensity of the colour was assessed with a spectrophotometer.

Specific antibodies to BoHV-1 were detected by INgezim IBR COMPAC 2.0 (n = 179) kits (Inmunologia y genetica aplicada). The test, based on the Blocking ELISA technique, is intended for cattle sera and milk samples. Plates are coated by a BoHV-1 antigen which binds the antibody in the serum. Briefly, after the first incubation and washing, the (peroxidase conjugated) monoclonal antibodies specific to the gB protein of BoHV-1 were added to each well and then incubated. The plates were then washed to remove non-fixed material. The substrate solution was then distributed to the wells, and plates were kept at room temperature in the dark. In the presence of peroxidase, a colorimetric reaction occurred. The stop solution was then added, and absorbance read with an EPOCH spectrophotometer. The intensity of the resulting yellow colour was inversely proportional to the degree of blocking by the conjugate, and hence the titre of specific antibodies in the samples.

INgezim Pestivirus COMPAC kits (Inmunologia y genetica aplicada) (n = 186) were used to detect specific antibodies to Ruminant Pestivirus. The test is based on blocking ELISA, and its mechanism is similar to INgezim IBR COMPAC 2.0. Pestivirus p80/p125 specific monoclonal antibodies coupled to peroxidase were used to bind free antigens fixed to the bottom of the wells: these bind any free antigens which remained unbound after the samples were added. Following the addition of peroxidase, the substrate undergoes a colorimetric reaction; as above, the presence of colour indicates a negative result.

Monoscreen Ab Elisa Bovine coronavirus (Bio-X Diagnostics S.A., Belgium) kits (n = 129) can be used to test sera, plasma, and colostrum for the presence of specific antibodies. The results can indicate whether a cow has had contact with pathogen, or whether a vaccine is effective. The microplates are coated with purified bovine coronavirus. In this procedure, serum samples and specific monoclonal antibody against coronavirus (peroxidase conjugated) were added to each well. After incubation and washing, a sensitive chromogen solution (TMB) was added. Stop solution was added and the blue colour changed to yellow. The intensity of the yellow colour was inversely proportionate to serum antibody titre.

### Statistical analysis

Statistical analysis was only performed for BTV, BoHV-1 and BCoV, as insufficient numbers of positive cases were found for the other pathogens. The frequency of occurrence of antibodies in European bison was analysed using generalized binary models, with the dependent variables being the presence of antibodies of given disease (marked as 1) and the absence of antibodies (marked as 0); these were tested in three separate models. The explanatory variables in all models were sex, age and study site. Study site was a grouping variable consisting of five groups (BIE: Bieszczady Mountains, BIA: Białowieska Forest, KNY: Knyszyńska Forest, BOR: Borecka Forest, ENC: animals in enclosures). We also compared pairwise means of BTV antibody occurrence with regard to study site using the Least Difference Test. Statistical significance was assumed at p < 0.05. The models were verified with ROC curve.

In the case of BTV, where age significantly increased the risk of seropositivity in animals, an additional analysis of the probability of seropositivity with age was performed using logistic regression. Four logistic regressions were performed: (A) for all examined European bison, (B) for European bison from the population with high BTV seroprevalence (Białowieska and Knyszyńska Forests), (C) for the European bison from the population with low BTV seroprevalence, (D) for the European bison from the enclosures. Similarly, the dependent variable was the presence or absence of BTV antibodies, but the explanatory variable was only age. Records which were suspect or with missing data (age or sex of animals) were excluded from the analysis. All statistical analyses were performed using IBM SPSS statistics software (version 28.0.1.0).

## Electronic supplementary material

Below is the link to the electronic supplementary material.

Supplementary Material 1

Supplementary Material 2

Supplementary Material 3

## Data Availability

All data and materials are available at the Department of Food Hygiene and Public Health Protection, Institute of Veterinary Medicine, Warsaw University of Life Sciences – SGGW, ul. Nowoursynowska 159, 02-776 Warsaw, Poland.

## Abbreviations

BCoV: bovine coronavirus
BIA: Białowieska Forest
BIE: Bieszczady Mountains
BoHV-1: Bovine herpesvirus-1
BRD: bovine respiratory disease
BOR: Borecka Forest
BT: bluetongue
BTV: bluetongue virus
BVDV: bovine viral diarrhea virus
ENC: animals in enclosures;
IBR: infectious bovine rhinotracheitis
IPV: pustular vulvovaginitis
IPB: infectious pustular balanoposthitis
IUCN: International Union for Conservation of Nature
KNY: Knyszyńska Forest
NT: near threatened
