# Supplementary material for: A serological survey of pathogens associated with the respiratory and digestive system in the Polish European bison (Bison bonasus) population in 2017–2022
Source: BMC Vet Res. 2023 Jun 1;19:74. doi: 10.1186/s12917-023-03627-y (PMC10233174; doi:10.1186/s12917-023-03627-y)
Supplement: Supplementary file 1 — Supplementary Material 1 [file 12917_2023_3627_MOESM1_ESM.docx]

Table S1. Effect of Study site, Sex and Age of animals on BTV antibodies occurrence in European bison in generalized linear binary model (BIE: Bieszczady Mountains, BIA: Białowieska Forest, KNY: Knyszyńska Forest, BOR: Borecka Forest, ENC: animals in enclosures), 0 – reference category.

| Source | B | SE | Wald χ^2^ | p | Exp (B) | Lower CI | Upper CI |
| --- | --- | --- | --- | --- | --- | --- | --- |
| Intercept | -4.507 | 0.6872 | -3.160 | <0.001 | 0.011 | 0.003 | 0.042 |
| Study Site (BIE) | -0.707 | 0.9874 | 1.229 | 0.474 | 0.493 | 0.071 | 3.416 |
| Study Site (BIA) | 3.163 | 0.7434 | 4.620 | <0.001 | 23.639 | 5.507 | 101,476 |
| Study Site (KNY) | 2.873 | 0.7193 | 4.283 | <0.001 | 17.697 | 4.321 | 72.479 |
| Study Site (BOR) | 1.160 | 0.8430 | 2.812 | 0.169 | 3.191 | 0.611 | 16.650 |
| Study Site (ENC) | 0 |  |  |  | 1 |  |  |
| Sex (F) | -0.143 | 0.4961 | .830 | 0.774 | 0.867 | 0.328 | 2.293 |
| Sex (M) | 0 |  |  |  | 1 |  |  |
| Age [years] | 0.161 | 0.0410 | .241 | <0.001 | 1.175 | 1.084 | 1.273 |
