# Supplementary material for: A serological survey of pathogens associated with the respiratory and digestive system in the Polish European bison (Bison bonasus) population in 2017–2022
Source: BMC Vet Res. 2023 Jun 1;19:74. doi: 10.1186/s12917-023-03627-y (PMC10233174; doi:10.1186/s12917-023-03627-y)
Supplement: Supplementary file 2 — Supplementary Material 2 [file 12917_2023_3627_MOESM2_ESM.docx]

Table S2. Effect of Study site, Sex and Age of animals on BoHV antibodies occurrence in European bison in generalized linear binary model (BIE: Bieszczady Mountains, BIA: Białowieska Forest, KNY: Knyszyńska Forest, BOR: Borecka Forest, ENC: animals in enclosures), 0 – reference category.

| Source | B | SE | Wald χ^2^ | p | Exp (B) | Lower CI | Upper CI |
| --- | --- | --- | --- | --- | --- | --- | --- |
| Intercept | 0.483 | 0.3330 | 2.102 | 0.147 | 1.621 | 0.844 | 3.112 |
| Study Site (BIE) | -0.160 | 0.5882 | 0.074 | 0.786 | 0.852 | 0.269 | 2.700 |
| Study Site (BIA) | 0.697 | 0.8225 | 0.718 | 0.397 | 2.007 | 0.400 | 10.062 |
| Study Site (KNY) | -0.282 | 0.5163 | 0.299 | 0.584 | 0.754 | 0.274 | 2.074 |
| Study Site (BOR) | 0.134 | 0.4961 | 0.073 | 0.788 | 1.143 | 0.432 | 3.022 |
| Study Site (ENC) | 0 |  |  |  | 1 |  |  |
| Sex (F) | 0.091 | 0.3533 | 0.067 | 0.796 | 1.095 | 0.548 | 2.189 |
| Sex (M) | 0 |  |  |  | 1 |  |  |
| Age [years] | -0.028 | 0.0388 | 0.528 | 0.467 | 0.972 | 0.901 | 1.049 |
