# Supplementary material for: A serological survey of pathogens associated with the respiratory and digestive system in the Polish European bison (Bison bonasus) population in 2017–2022
Source: BMC Vet Res. 2023 Jun 1;19:74. doi: 10.1186/s12917-023-03627-y (PMC10233174; doi:10.1186/s12917-023-03627-y)
Supplement: Supplementary file 3 — Supplementary Material 3 [file 12917_2023_3627_MOESM3_ESM.docx]

Table S3. Effect of Study site, Sex and Age of animals on Coronavirus antibodies occurrence in European bison in generalized linear binary model (BIE: Bieszczady Mountains, BIA: Białowieska Forest, KNY: Knyszyńska Forest, BOR: Borecka Forest, ENC: animals in enclosures), 0 – reference category.

| Source | B | SE | Wald χ^2^ | p | Exp (B) | Lower CI | Upper CI |
| --- | --- | --- | --- | --- | --- | --- | --- |
| Intercept | -1.372 | 0.4509 | 9.257 | 0.002 | 0.254 | 0.105 | 0.614 |
| Study Site (BIE) | 1.242 | 0.5997 | 4.292 | 0.038 | 3.464 | 1.069 | 11.220 |
| Study Site (BIA) | 0.697 | 0.8849 | 0.620 | 0.431 | 2.007 | 0.354 | 11.371 |
| Study Site (KNY) | 0.032 | 0.5767 | 0.003 | 0.955 | 1.033 | 0.334 | 3.198 |
| Study Site (BOR) | -0.223 | 0.7276 | 0.094 | 0.760 | 0.800 | 0.192 | 3.332 |
| Study Site (ENC) | 0 |  |  |  | 1 |  |  |
| Sex (F) | 0.239 | 0.4421 | 0.293 | 0.589 | 1.270 | 0.534 | 3.021 |
| Sex (M) | 0 |  |  |  | 1 |  |  |
| Age [years] | -0.004 | 0.0424 | 0.007 | 0.932 | 0.996 | 0.917 | 1.083 |
